# Dihydromyricetin Alleviates Fermented Rapeseed Meal-Induced Intestinal Injury in Chinese Soft-Shelled Turtle (*Pelodiscus sinensis*): Insights from Growth, Antioxidant, Inflammatory, Transcriptomic and Metabolomic Assessments

**DOI:** 10.3390/antiox15070856

**Published:** 2026-07-08

**Authors:** Wenshu Liu, Lun Chen, Wencai Liu, Jingjing Lu, Yuzhu Wang, Xiaoze Guo, Lingya Li, Xu Han, Chuang Mei, Siming Li, Zirui Wang

**Affiliations:** 1 College of Animal Science and Technology, Jiangxi Agricultural University, Nanchang 330045, China; liuws@jxaas.cn (W.L.); 19880007585@163.com (L.C.); 2 Institute of Animal Husbandry and Veterinary, Jiangxi Academy of Agricultural Sciences, Nanchang 330200, China; lujingjing@jxaas.cn (J.L.); wangyuzhu@jxaas.cn (Y.W.); guoxz@jxaas.cn (X.G.); lilingya@jxaas.cn (L.L.); 1985978780@163.com (X.H.); mei_chuang@163.com (C.M.); 3 Jiangxi Province Key Laboratory of Animal Green and Healthy Breeding, Nanchang 330200, China; 4 Nanfeng County Bureau of Agriculture and Rural Affairs, Fuzhou 344500, China; nfzj2011@163.com

**Keywords:** dihydromyricetin, Chinese soft-shelled turtle, intestinal mucosal oxidative damage, transcriptomic, metabolomic

## Abstract

This study was designed to assess the protective efficacy of dihydromyricetin (DHM) against intestinal injury in Chinese soft-shelled turtles (*Pelodiscus sinensis*) caused by partial replacement of fishmeal (FM) with fermented rapeseed meal (FRM), and to uncover the molecular mechanisms involved. Turtles were fed an FM-based control diet, an FRM-substituted diet, or FRM diets supplemented with DHM at 0.5‰ (DHMT1), 1.0‰ (DHMT2) or 2.0‰ (DHMT3) for 8 weeks. Growth performance, digestive enzyme activities, antioxidant parameters, inflammatory cytokines, and intestinal histomorphology were assessed, and intestinal transcriptomics and metabolomics were also performed. FRM replacement significantly improved growth performance; however, this beneficial effect was accompanied by notable intestinal mucosal oxidative damage, as evidenced by decreased villus height, increased lumen space, and lamina propria edema, along with elevated pro-inflammatory cytokines (IL-1β and TNF-α) and MDA content, alongside reduced GSH and CAT activities. DHM supplementation dose-dependently ameliorated these adverse effects by restoring mucosal integrity, digestive enzyme activities, redox homeostasis, and inflammatory balance, ultimately improving growth performance. Transcriptomic KEGG analysis revealed that DHM enriched pathways related to glycerophospholipid metabolism, glutathione metabolism, drug metabolis-cytochrome P450, and polyunsaturated fatty acid metabolism. Metabolomics further confirmed dose-dependent remodeling of phospholipids and bile acids. Integrated omics demonstrated that DHM likely regulates detoxification, anti-inflammatory, membrane repair, and antioxidant pathways. In conclusion, DHM demonstrates a protective effect against FRM-induced intestinal mucosal oxidative damage in *P. sinensis*, which may be mediated by a synergistic combination of enhanced detoxification, anti-inflammatory modulation, restoration of phospholipid membrane integrity, and reinforcement of antioxidant defenses.

## 1. Introduction

In recent years, the global aquaculture industry has faced mounting pressure from the steadily rising cost of feed, a primary driver of which is the increasing price of imported fish meal (FM). To mitigate expenses, feed producers have increasingly turned to partial replacement of FM with more affordable plant-based protein sources [1,2]. While cost-effective, this practice has been frequently associated with compromised immune function in cultured aquatic species, leading to a higher incidence of enteritis compared to previous years [3,4,5]. The long-term and excessive application of chemical therapeutics and antibiotics can severely damage the intestinal mucosal immune system of fish, further diminishing their disease resistance and implicating food safety [6]. Consequently, there is growing and urgent interest in identifying natural anti-inflammatory substances as potential therapeutic agents or feed additives for preventing and managing enteritis in aquaculture [7,8].

Plant extracts, rich in bioactive compounds, represent a promising frontier in novel feed additive development. These extracts can modulate both specific and non-specific intestinal immunity, thereby enhancing overall gut health and immune competence [9,10,11]. *Ampelopsis grossedentata*, commonly known as vine tea or “Tengcha” is an ancient herbal, tea-like, and medicinal food homologous plant resource. Vine tea is exceptionally rich in active compounds, with total flavonoid content reaching up to 43% of its dry weight. Dihydromyricetin (DHM) constitutes approximately 30% of this flavonoid content, making it the predominant active ingredient [12]. In commercial practice, vine tea is prized for and produced solely from its delicate shoot tips. Consequently, a substantial portion of the plant biomass is treated as agricultural by-products, leading to considerable resource underutilization [13].

Modern pharmacological studies have revealed that DHM possesses a wide spectrum of biological activities, including anti-inflammatory, analgesic, antimicrobial, antioxidant, antitumor, hepatoprotective, hypolipidemic, hypoglycemic, and immunomodulatory effects, marking it as an ideal candidate for a novel antioxidant feed additive [14,15]. Furthermore, the safety profile of vine tea and its extracts has been validated. In rats, dihydromyricetin (DHM)-rich herbal mixture extracts had no subacute toxicity on liver and renal function in assay of 28 days of repeated feeding study [16]. In a long-term (12 weeks) toxicity model in rats, various doses of vine tea total flavonoids showed no significant differences compared to the blank control group in terms of general appearance, behavior, body weight, organ coefficients, hematological, and biochemical indices. Pathological examinations revealed no obvious lesions related to drug toxicity, and no delayed toxic reactions were observed after drug withdrawal [17]. In an aquaculture study, plasma transaminase levels and hepatopancreas microscopic observations indicated that dietary DHM (100, 200, 300 mg/kg) did not cause damage to the hepatopancreas of shrimp (*Litopenaeus vannamei*) after a 56-day feeding trial [18].

In recent years, the effects and mechanisms of DHM in terrestrial livestock have been increasingly reported [19,20]. However, to the best of our knowledge, research on DHM in aquatic animals remains relatively scarce. Dihydromyricetin (DHM) has garnered attention for its broad-spectrum beneficial effects in several cultured aquatic species, including large yellow croaker (*Larimichthys crocea*) [21], *Megalobrama hoffmanni* [22], *Mylopharyngodon piceus* [23], and shrimp (*L. vannamei*) [18,24]. Research indicates that DHM supplementation can effectively promote growth performance, enhance antioxidant capacity and non-specific immunity, improve intestinal health and flesh quality, and ultimately strengthen disease resilience.

Despite the multiple beneficial effects of DHM demonstrated in terrestrial animals, fish, and shrimp, no study has yet reported its effects on reptiles such as the Chinese soft-shelled turtle (*P. sinensis*). The Chinese soft-shelled turtle is an important source of high-quality protein in China, highly prized by consumers for its exceptional nutritional value and unique flavor, leading to steadily growing market demand. In 2024, its production reached 541,589 tons [25]. The high fishmeal content in turtle feed has led to continuously rising feed prices, which has a significant impact on the industry. In our previous study, we found that replacing 10% fish meal with fermented rapeseed meal induced intestinal inflammation. Therefore, this study aims to evaluate the intestinal health-promoting effects of DHM on Chinese soft-shelled turtles (*P. sinensis*) fed a basal diet where fish meal was partially replaced by fermented rapeseed meal, and to analyze the related mechanisms from the perspectives of changes in the transcriptome and metabolome. It seeks not only to provide a novel option for a gut-health protectant under fishmeal replacement strategies but also to offer critical applied evidence and data support for addressing the issue of massive by-product waste in the vine tea industry and facilitating their high-value utilization.

## 2. Materials and Methods

### 2.1. Diets

A total of five dietary treatments were formulated in this experiment, with one specific diet per treatment group. These included: the fish meal control group (containing 50% fish meal, labeled as FM), the fermented rapeseed meal replacement group (10% fish meal replaced by fermented rapeseed meal, labeled as FRM), and three DHM supplementation groups based on the fermented rapeseed meal replacement diet with graded DHM levels at 0.5‰ (DHMT1), 1.0‰ (DHMT2), and 2.0‰ (DHMT3). The diets were formulated using fish meal as the primary protein source, wheat flour and α-starch as the main carbohydrate sources, and fish oil and soybean oil as the primary lipid sources. The formulation and proximate chemical composition of the basal and experimental diets are detailed in Table S1. All raw ingredients were ground to pass through a 60-mesh sieve, thoroughly mixed, and then processed into pellets using a twin-screw extruder. The pellets were air-dried naturally for 12 h, subsequently packed in sealed bags, and stored at −20°C until use.

### 2.2. Animal Rearing Management

The experimental Chinese soft-shelled turtles were purchased from a farm in Nanfeng County, Fuzhou City, Jiangxi Province. A total of 300 turtles with strong vitality and similar size (average body weight: approximately 500 g) were randomly selected and allocated into 15 culture ponds, with 20 turtles per pond. Each pond has an area of 80 m^2^ and a depth of 1 m. Each diet was randomly assigned to three ponds. The turtles were fed to apparent satiation twice daily (at 06:30 and 17:30), with the feeding amount adjusted to ensure no visible feed remnants remained on the water surface after 40 min. Daily pond inspections were conducted twice to observe feeding behavior, growth status, and to adjust feeding rations accordingly. Records were kept throughout the culture period regarding feed administration, turtle activity, and mortality. The water quality parameters were monitored every three days in the morning using a portable multi-parameter water quality analyzer (Octadem Model W-II, Octadem, Wuxi, China) following the manufacturer’s instructions. Over the 8-week experimental period, the water quality remained within the following ranges: temperature 26.2~32.8°C, dissolved oxygen 6.7~7.0 mg/L, pH 6.7~7.3, ammonia nitrogen 0.23~0.51 mg/L, and nitrite nitrogen 0.01~0.02 mg/L.

### 2.3. Growth Assessment and Sampling

At the end of the feeding trial, all turtles were fasted for 24 h, captured, counted, and weighed per replicate. Subsequently, four turtles were randomly sampled from each replicate. Intestinal tissues were dissected. After being rinsed clean with PBS solution, the intestine was roughly divided into three segments of equal length. The proximal segment was used for the analysis of digestive enzyme activities, the middle segment was fixed in 4% paraformaldehyde for mucosal structure analysis, and the distal segment was snap-frozen in liquid nitrogen and then transferred to a −80 °C freezer for subsequent transcriptomic and metabolomic analyses, and detection of antioxidant and nonspecific immune indicators.

### 2.4. Determination of Intestinal Digestive and Antioxidant Enzyme Activities

The activities of intestinal total protein (TP), Trypsin, amylase, as well as glutathione peroxidase (GSH-Px), catalase (CAT), superoxide dismutase (SOD), and the content of malondialdehyde (MDA) were determined using commercial assay kits (manufactured by the Nanjing Jiancheng Bioengineering Institute, Nanjing, China) strictly following the manufacturer’s protocols. Three intestinal tissue samples were randomly collected from each treatment group, with three technical replicates per sample.

### 2.5. Evaluation of Intestinal Cytokine Levels

The levels of IL-1β, TNF-α, TGF-β, and IL-10 in the intestinal homogenate supernatant of *P. sinensis* were determined using ELISA kits purchased from Nanjing Jiancheng Bioengineering Institute. The specific measurement methods and operational procedures were carried out in accordance with the manufacturer’s instructions. After adding the samples as described in the protocol, the absorbance values were measured using a microplate reader, and the levels of each indicator were calculated according to the corresponding formulas. Three intestinal tissue samples were randomly collected from each treatment group, with three technical replicates per sample.

### 2.6. Histomorphological Examination of Intestinal Mucosa

Intestinal tissue samples fixed in 4% paraformaldehyde were dehydrated through a graded ethanol series, embedded in paraffin, and sectioned. Five fixed samples of each treatment were randomly selected and dried in a 37 °C incubator, stained with hematoxylin and eosin (H&E), and finally observed and photographed using an Olympus microscope (Olympus Corporation, Tokyo, Japan, model DP72). Villus height was measured using Saiviewer software (Servicebio, Wuhan, China, https://www.servicebio.com/data-detail?code=RJXZ&id=5139 accessed on 1 May 2026).

### 2.7. Transcriptomic Analysis of Intestine

Five intestine samples of each treatment were randomly selected and sent to Wuhan Metware Company for transcriptome sequencing and analysis. Total RNA was isolated from intestine samples using TRIzol reagent (Thermo Fisher Scientific, Waltham, MA, USA), and its quality was verified by electrophoresis, spectrophotometry, and bioanalyzer. Sequencing libraries were then prepared from qualified RNA samples using the NEBNext^®^ Ultra™ RNA Library Prep Kit (New England Biolabs, Inc., Ipswich, MA, USA). Indexed libraries were clustered on an Illumina cBot system with the TruSeq PE Cluster Kit (Illumina, San Diego, CA, USA) under the following thermal conditions: 98 °C for 30 s; 10 cycles of 98 °C for 10 s, 60 °C for 30 s, and 72 °C for 30 s; and a final 72 °C for 5 min. The flow cell was then transferred to an Illumina sequencer for 150 bp paired-end sequencing, maintaining a Q30 score above 80%. Real-time base calling converted raw image data to FASTQ files. Clean reads were obtained by filtering raw data with fastp, mapping to the reference genome using HISAT2 v2.2.1 (https://daehwankimlab.github.io/hisat2/ accessed on 1 May 2026), and analyzing for differential expression with DESeq2 v1.52.0 (https://github.com/thelovelab/DESeq2 accessed on 1 May 2026). Transcripts meeting the criteria of |log_2_(fold change)| ≥ 1 and an adjusted *p*-value (FDR) ≤ 0.05 were defined as significantly differentially expressed genes.

To verify the RNA-seq results, RT-qPCR was performed to examine the expression of the following genes: *RBPJL*, *FBXL22*, *ITGA5*, *SYNPO2L*. Total RNA was extracted from three turtles per treatment using the TRIzon^®^ Reagent Up Plus RNA Kit (CWBIO, Shanghai, China) following the manufacturer’s protocol. Reverse transcription was carried out with the NovoScript^®^ Plus All-in-one 1st Strand cDNA Synthesis SuperMix (gDNA Purge) (Novoprotein, Shanghai, China) as instructed. Specific qPCR primers (Table S2) were carefully designed based on sequences retrieved from GenBank using Primer 5 software (Premier Biosoft, Palo Alto, CA, USA). Quantitative PCR was performed in a CFX Connect™ Real-Time PCR instrument (Bio-Rad Laboratories, Shanghai, China) using Novostart^®^ Universal Fast SYBR qPCR SuperMix (Novoprotein Scientific Inc., Suzhou, China). Each 20 μL reaction contained 10 μL ChamQ Universal SYBR qPCR Master Mix (2×), 1 μL cDNA, 0.4 μL each of forward and reverse primers, and 8.2 μL ddH_2_O. The thermal cycling conditions were 95 °C for 10 min, followed by 40 cycles of 95 °C for 10 s, 60 °C for 30 s, and 72 °C for 30 s. All reactions were run in triplicate. β-actin was used as the internal reference gene, and relative gene expression was calculated using the 2^−ΔΔCT^ method. In addition, we also examined the expression levels of *NLRP3*, *CYP8B1*, *DUOXA2*, and *DUOX2*, which are key genes implicated in antioxidant defense, inflammatory pathways, lipid metabolism, and cytochrome P450-mediated detoxification.

### 2.8. Metabolome Analysis of Intestine

For the untargeted metabolomic analysis, five frozen samples (−80 °C) of each treatment were homogenized on ice and extracted with 400 μL of cold methanol/water (7:3, *v*/*v*) containing internal standards. After vortexing, incubation, and centrifugation at 4 °C, the supernatant was freeze-precipitated at −20 °C and re-centrifuged. A 200 μL aliquot of the final supernatant was injected for LC-MS analysis (Thermo Scientific, Massachusetts, MA, USA). Chromatographic separation was performed on a Waters ACQUITY Premier HSS T3 Column (40 °C, Waters Corporation, Milford, MA, USA) using a gradient of 0.1% formic acid in water (A) and acetonitrile (B) at 0.4 mL/min: 5% to 20% B in 2 min, to 60% B in 3 min, to 99% B in 1 min (hold 1.5 min), and re-equilibration to 5% B. Mass spectrometry was conducted in IDA mode on a TripleTOF system (SCIEX, Framingham, MA, USA). The ESI source was set to 550 °C with GAS1/GAS2 at 50 psi, CUR at 25 psi, and ion spray voltages of ±5000/–4000 V. Full-scan spectra (50–1000 Da, 200 ms) and MS/MS spectra (25–1000 Da, 40 ms) were acquired using collision energies of ±30 V. Raw data were converted to mzML format and processed with XCMS for peak detection, alignment, and retention time correction. Peak areas were normalized (SVR algorithm), and features not present in >50% of samples per group were removed. Metabolites were putatively identified by matching to databases. Differential metabolites were screened using VIP > 1.0 (from OPLS-DA models) and a *t*-test *p* < 0.05, followed by KEGG annotation and pathway enrichment analysis via a hypergeometric test.

### 2.9. Statistical Analysis

In this study, except for transcriptomic and metabolomic data, the remaining data were subjected to one-way analysis of variance (ANOVA), followed by Tukey’s multiple comparisons test to evaluate intergroup differences. Results were visualized using GraphPad Prism 9.5 (GraphPad Software, San Diego, CA, USA). All results are presented as means ± standard error of the mean (SEM), and *p*  <  0.05 was considered statistically significant.

## 3. Results

### 3.1. Growth Performance

The growth performance of *P. sinensis* across different treatment groups is presented in Table S3. The final body weight in the FRM group was comparable to that of the FM group, whereas the DHMT2 and DHMT3 groups exhibited significantly higher final body weights than the FM and FRM groups (*p* < 0.05). The weight gain rate and specific growth rate in the FRM group were significantly higher than those in the FM group, and both parameters increased in a dose-dependent manner following DHM supplementation, with the DHMT3 group achieving the highest values (*p* < 0.05). The feed conversion ratio in the FRM group was significantly lower than those in the FM and DHMT1 groups, as well as in DHMT2 and DHMT3 groups (*p* < 0.05). No significant differences were observed in the viscerosomatic index, hepatosomatic index, or calipash ratio among any of the treatment groups. These results suggest that FRM replacement of fishmeal significantly improved growth performance and feed efficiency, and that DHM supplementation further enhanced these effects in a dose-dependent manner.

### 3.2. Intestinal Digestive Enzyme Activities and Antioxidant Parameters

The intestinal digestive enzyme activities and antioxidant parameters are presented in Table 1. No significant difference was observed in total protein content in the intestine of *P. sinensis* among the treatment groups. Regarding digestive enzymes, amylase activity in the fermented rapeseed meal replacement group (FRM) was significantly lower than that in the DHMT1 group (*p* < 0.05), while no significant differences were found among the other groups (*p* > 0.05). Trypsin activity in the DHMT2 and DHMT3 groups was significantly higher than that in the other three groups (*p* < 0.05).

In terms of antioxidant indicators, glutathione (GSH) and catalase (CAT) activities in the FRM group were lower than those in the FM group, but were significantly restored or even exceeded FM levels in the DHM-supplemented groups (*p* < 0.05). Notably, CAT activity in the DHMT2 and DHMT3 groups was significantly higher than that in all other groups (*p* < 0.05). Interestingly, superoxide dismutase (SOD) activity showed inconsistent trends across groups, with the FRM group exhibiting higher activity than the FM group (*p* < 0.05), though the differences were not statistically significant compared to other groups (*p* > 0.05). Meanwhile, malondialdehyde (MDA) content, which reflects the degree of lipid peroxidation, was significantly reduced to the lowest level in the DHMT3 group (*p* < 0.05).

### 3.3. Levels of Intestinal Cytokines

**Figure 1:**
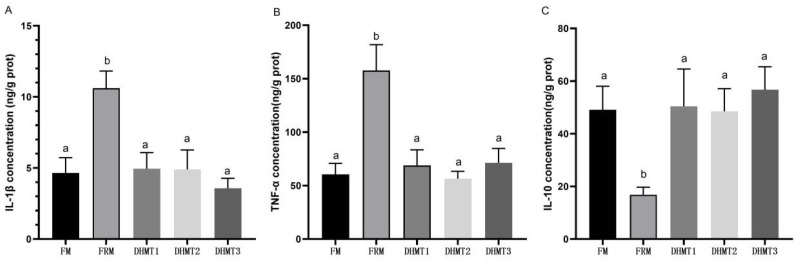
The intestinal cytokine levels of *P. sinensis* in different treatments (*n* = 3). (**A**) IL-1β concentration; (**B**) TNF-α concentration; (**C**) IL-10 concentration. Note: FM, fish meal; FRM, fermented rapeseed meal replaced; DHMT1/2/3, FRM + 0.5/1.0/2.0‰ DHM. Different letters in the same row indicate significant differences (*p* < 0.05).

The results of inflammatory cytokine levels in the intestinal tissue of *P. sinensis* are presented in Figure 1. Compared with the baseline levels of IL-1β and TNF-α in the FM group, the concentrations of both pro-inflammatory cytokines were significantly increased in the FRM group (*p* < 0.05). Following DHM supplementation, IL-1β and TNF-α levels were significantly reduced in all DHM-treated groups (*p* < 0.05). In contrast, the levels of the anti-inflammatory cytokine IL-10 exhibited an opposite trend. These results indicate that FRM replacement significantly upregulated the expression of pro-inflammatory cytokines, and DHM supplementation effectively reversed this elevation.

### 3.4. Changes in the Morphology of the Intestinal Mucosa

**Figure 2:**
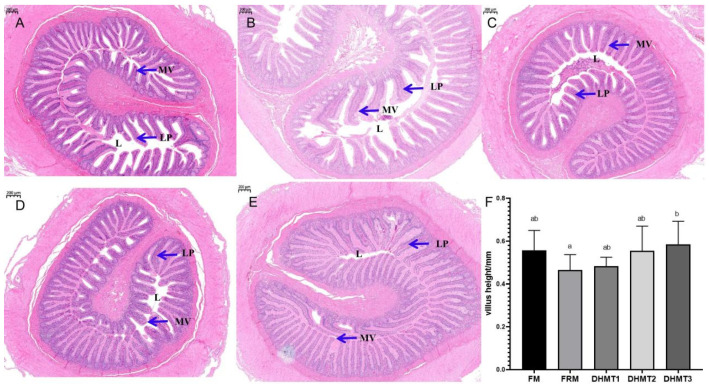
The intestinal mucosal morphology of *P. sinensis* in different treatments (*n* = 3). (**A**) FM group; (**B**) FRM group; (**C**) DHMT1 group; (**D**) DHMT2 group; (**E**) DHMT3 group; (**F**) villus height of different groups. Note: FM, fish meal; FRM, fermented rapeseed meal replaced; DHMT1/2/3, FRM + 0.5/1.0/2.0‰ DHM. Different letters in the same row indicate significant differences (*p* < 0.05). MV, microvillus; LP, lamina propria. L, lumen.

The results of H&E staining of the intestinal mucosa of *P. sinensis* are shown in Figure 2. Following the replacement of fishmeal with fermented rapeseed meal, the intestinal lumen space increased, and the lamina propria exhibited mild edema. After DHM supplementation, the lumen space decreased, and the morphology of the villi and lamina propria was restored to that of the FM group. Statistical analysis of intestinal villus height revealed a decreasing trend after the replacement of fishmeal with fermented rapeseed meal. Following DHM supplementation, villus height gradually increased, with the high-dose DHMT3 group showing significantly higher villus height than the FRM group (*p* < 0.05).

### 3.5. Changes of the Intestinal Transcriptome

#### 3.5.1. Annotation and Function Analysis

The number of raw sequences obtained per sample ranged from 43,930,048 to 67,645,086, with data sizes between 6.59 Gb and 10.15 Gb. After filtering, the clean reads ranged from 43,392,466 to 65,898,940, corresponding to data sizes between 6.51 Gb and 9.88 Gb. The overall sequencing error rate remained low at 0.02%. The proportion of bases achieving Qphred scores of at least 20 (Q20) and 30 (Q30) reached 99.47% and 98.21%, respectively, reflecting high base-calling accuracy. Furthermore, the GC content across samples ranged from 44.05% to 48.66% (Table S4).

Upon acquisition of clean data, alignment was performed against the reference genome of *P. sinensis*. The resulting mapping statistics, including the total number of reads aligned to the genome, the count uniquely mapped, and those aligned to multiple positions, are summarized in Table S5. All samples demonstrated high mapping efficiency, with over 86.70% of reads aligned to the reference and more than 84.43% uniquely mapped.

#### 3.5.2. Differential Analysis

**Figure 3:**
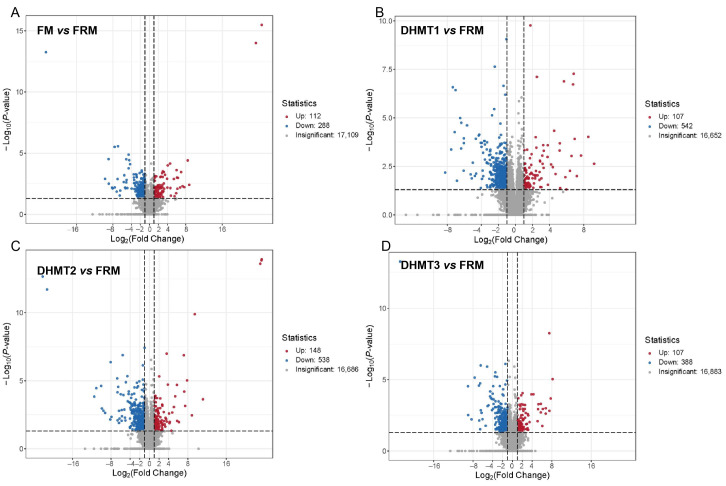
Volcano Plot of Differentially Expressed Genes in the Intestine of *P*. *sinensis* (*n* = 5). (**A**) differentially expressed genes between FM and FRM group; (**B**) differentially expressed genes between DHMT1 and FRM group; (**C**) differentially expressed genes between DHMT2 and FRM group; (**D**) differentially expressed genes between DHMT3 and FRM group. Note: FM, fish meal; FRM, fermented rapeseed meal replaced; DHMT1/2/3, FRM + 0.5/1.0/2.0‰ DHM. *p* ≤ 0.05 were defined as significantly differentially expressed genes.

Volcano plots illustrating the differentially expressed genes (DEGs) in the intestine of *P. sinensis* between the FRM and FM groups, as well as between DHM-treated groups and the FRM group, are shown in Figure 3. A total of 400 DEGs were identified between the FRM and FM groups, comprising 112 upregulated and 288 downregulated transcripts (Figure 3A). Compared to the FRM group, 649 genes showed significant differential expression in the DHMT1 group, including 107 upregulated and 542 downregulated genes. In the DHMT2 group, 686 genes were significantly altered, with 148 upregulated and 538 downregulated. Meanwhile, 495 genes were significantly changed in the DHMT3 group, consisting of 107 upregulated and 388 downregulated transcripts (Figure 3B–D).

**Figure 4:**
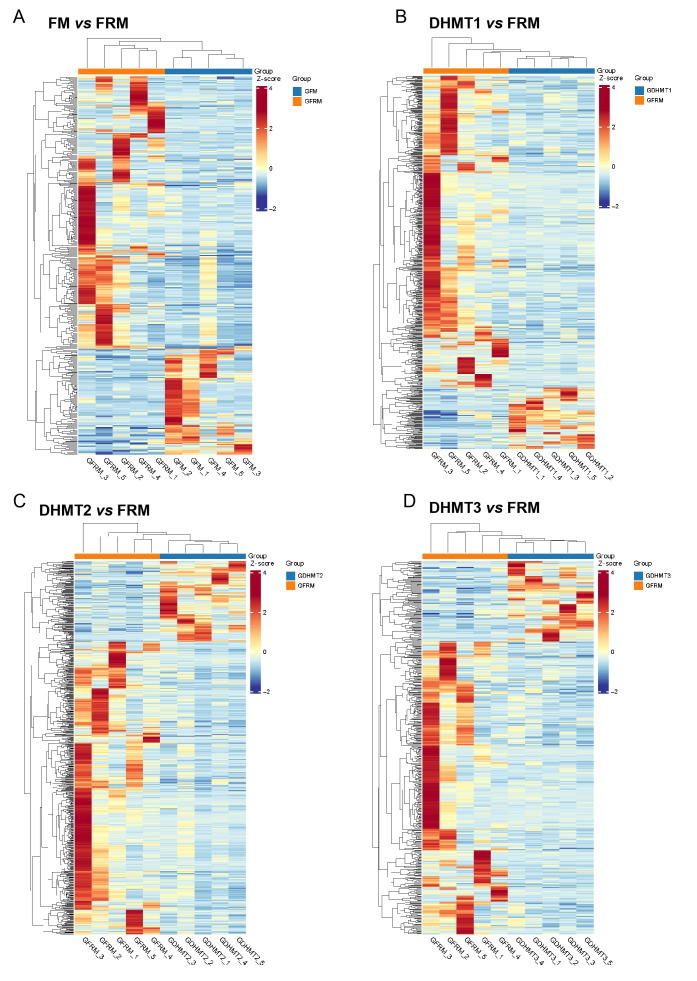
Heatmap of DEGs in the Intestine of *P. sinensis* (*n* = 5). (**A**) clustering of differentially expressed genes between FM and FRM group; (**B**) clustering of differentially expressed genes between DHMT1 and FRM group; (**C**) clustering of differentially expressed genes between DHMT2 and FRM group; (**D**) clustering of differentially expressed genes between DHMT3 and FRM group. Note: FM, fish meal; FRM, fermented rapeseed meal replaced; DHMT1/2/3, FRM + 0.5/1.0/2.0‰ DHM.

A clustering heatmap of DEGs in the soft-shelled turtle intestine samples is presented in Figure 4. The cluster analysis revealed a distinct expression pattern in the FRM group compared to the FM group, as well as between DHM-treated groups and the FRM group.

**Figure 5:**
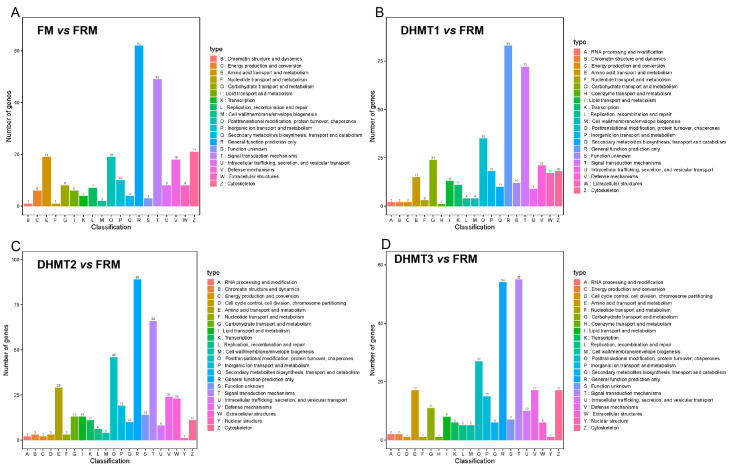
KOG Annotation of Differentially Expressed Genes in the Intestine of *P. sinensis* (*n* = 5). (**A**) KOG annotation of differentially expressed genes between FM and FRM group; (**B**) KOG annotation of differentially expressed genes between DHMT1 and FRM group; (**C**) KOG annotation of differentially expressed genes between DHMT2 and FRM group; (**D**) KOG annotation of differentially expressed genes between DHMT3 and FRM group. Note: FM, fish meal; FRM, fermented rapeseed meal replaced; DHMT1/2/3, FRM + 0.5/1.0/2.0‰ DHM.

Figure 5 illustrates the KOG (Eukaryotic Orthologous Groups) functional classification of DEGs in the intestines of Chinese soft-shelled turtles across different treatment groups. The DEGs were functionally annotated against the KOG (Eukaryotic Orthologous Groups) database. A total of 23 well-defined functional categories were identified in the intestinal tissue (excluding those classified as general function prediction only or unknown function). The bar chart displays the number of genes enriched in each functional category for the experimental groups (GFM, GFRM, GDHMT1, GDHMT2, GDHMT3). The results indicate that the DEGs were predominantly enriched in functions related to “Signal transduction mechanisms”, “Posttranslational modification, protein turnover, chaperones”, “Carbohydrate transport and metabolism”, “Amino acid transport and metabolism”, “Defense mechanisms” and “Extracellular structures”.

#### 3.5.3. KEGG Analysis

**Figure 6:**
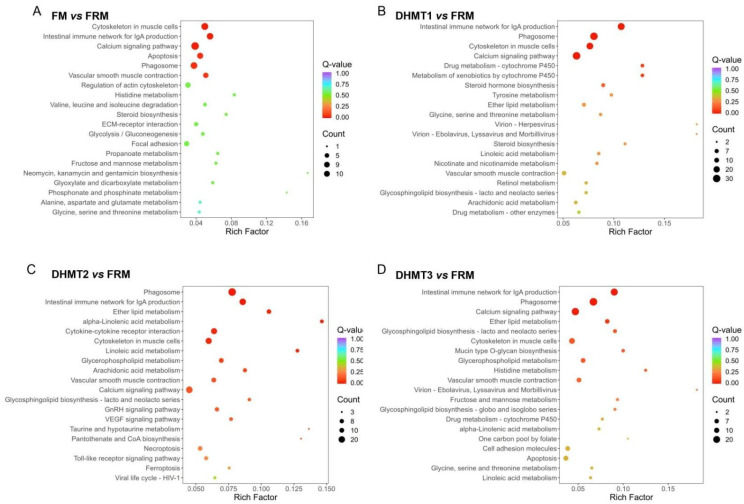
KEGG enrichment sites of differentially expressed genes in the Intestine of *P. sinensis* (TOP 20) (*n* = 5). (**A**) KEGG enrichment of differentially expressed genes between FM and FRM group; (**B**) KEGG enrichment of differentially expressed genes between DHMT1 and FRM group; (**C**) KEGG enrichment of differentially expressed genes between DHMT2 and FRM group; (**D**) KEGG enrichment of differentially expressed genes between DHMT3 and FRM group. Note: FM, fish meal; FRM, fermented rapeseed meal replaced; DHMT1/2/3, FRM + 0.5/1.0/2.0‰ DHM.

Figure 6 displays the top 20 significantly enriched KEGG (Kyoto Encyclopedia of Genes and Genomes) pathways for the DEGs identified in the turtle intestine. Compared with the FRM group, the differentially expressed genes in the FM group were predominantly enriched in pathways related to structural integrity and basic metabolism. The most enriched pathways included “Cytoskeleton in muscle cells”, “Intestinal immune network for IgA production”, “Calcium signaling pathway”, “Apoptosis”, and “Phagosome”. Additionally, multiple metabolic pathways related to amino acid degradation (e.g., “Valine, leucine, and isoleucine degradation”, “Glycine, serine, and threonine metabolism”) and carbohydrate metabolism (e.g., “Glycolysis/Gluconeogenesis”, “Fructose and mannose metabolism”) were also significantly enriched. Following low-dose DHM supplementation (DHMT1), the immune-related pathways such as “Intestinal immune network for IgA production” and “Phagosome” remained enriched. But the enriched pathways shifted towards detoxification and specific lipid metabolism. Key enriched pathways included “Drug metabolism-cytochrome P450”, “Metabolism of xenobiotics by cytochrome P450”, and “Steroid hormone biosynthesis”. Furthermore, pathways involved in fatty acid metabolism, including “Linoleic acid metabolism” and “Arachidonic acid metabolism”, began to appear. With medium-dose DHM supplementation (DHMT2), the enrichment pattern showed a strong focus on inflammatory signaling and fatty acid metabolism. Notably, “Cytokine–cytokine receptor interaction”, “Toll-like receptor signaling pathway”, and “Necroptosis” were significantly enriched, suggesting modulation of immune signaling. Lipid metabolism pathways became more prominent, including “Alpha-Linolenic acid metabolism”, “Linoleic acid metabolism”, “Arachidonic acid metabolism”, and “Glycerophospholipid metabolism”. Additionally, “Ferroptosis”, a regulated cell death pathway linked to lipid peroxidation, was also enriched. In the high-dose DHM group (DHMT3), the enriched pathways demonstrated a combination of immune modulation and enhanced metabolic repair. “Intestinal immune network for IgA production”, “Phagosome,” and “Apoptosis” were enriched, indicating ongoing immune regulation. Detoxification pathways such as “Drug metabolism-cytochrome P450” re-emerged. Crucially, several pathways related to one-carbon metabolism and redox balance, including “One carbon pool by folate” and “Glycine, serine and threonine metabolism” were significantly enriched.

#### 3.5.4. qPCR Validation

The expression levels of four genes (RBPJL, FBXL22, ITGA5, SYNPO2L4) in the intestine were validated by RT-qPCR (Figure S1). The expression patterns of these genes were consistent with the upregulation and downregulation trends observed in RNA-seq analysis. The transcript levels of *NLRP3, CYP8B1, DUOXA2*, and *DUOX2* are presented in Figure S2. In the FRM group, the expression of the inflammatory gene NLRP3 was significantly elevated compared with the other treatment groups. Meanwhile, the genes associated with oxidative detoxification, DUOXA2 and DUOX2, were also markedly upregulated, whereas CYP8B1 expression was significantly reduced.

### 3.6. Changes in the Intestinal Metabolic Profile

#### 3.6.1. General Overview of Intestinal Metabolites

**Figure 7:**
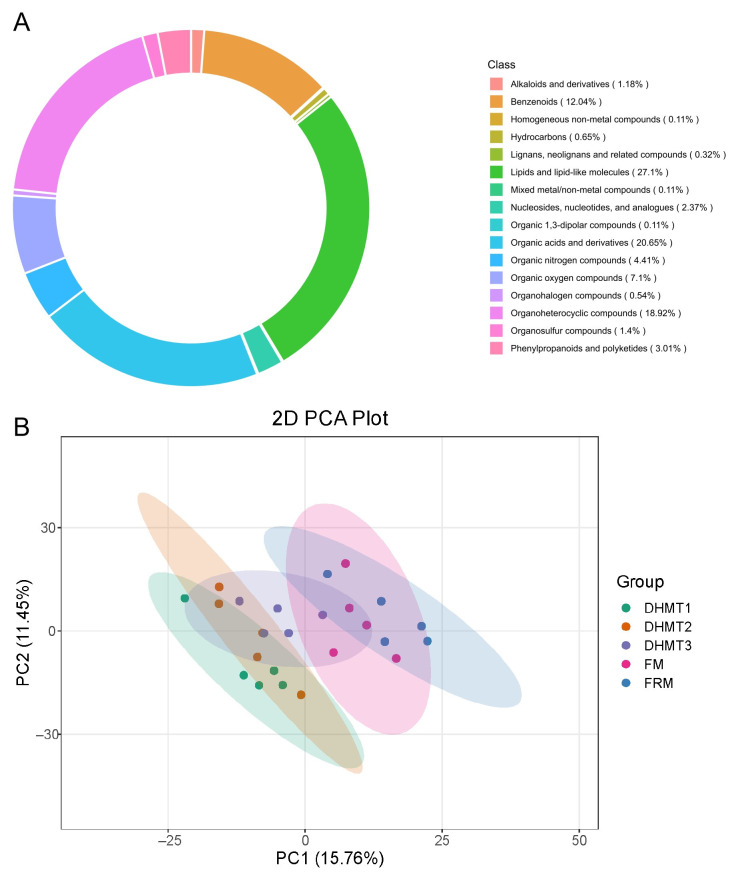
Circular graph of the composition of intestinal metabolites of *P*. *sinensis* (**A**) and principal component analysis (**B**) (*n* = 5). Note: FM, fish meal; FRM, fermented rapeseed meal replaced; DHMT1/2/3, FRM + 0.5/1.0/2.0‰ DHM.

A total of 1780 metabolites were detected from all samples in this experiment, with 1561 of them confirmed at the secondary identification level. Among these, 833 metabolites were detected in the positive ion mode of mass spectrometry (MS), while 947 were detected in the negative ion mode. The circular diagram depicting the composition of metabolite categories is presented in Figure 7A. A Principal Component Analysis (PCA) score plot showing the distribution of all samples in a two-dimensional space, where each point represents a single sample. The PCA plot revealed clear clustering and separation among the experimental groups (FM, FRM, DHMTs). Notably, the FRM group clusters distinctly away from the GFM control group, while the DHM-treated groups are positioned intermediately or closer to the FM group (Figure 7B).

#### 3.6.2. Differential Metabolites Among Different Treatments

**Figure 8:**
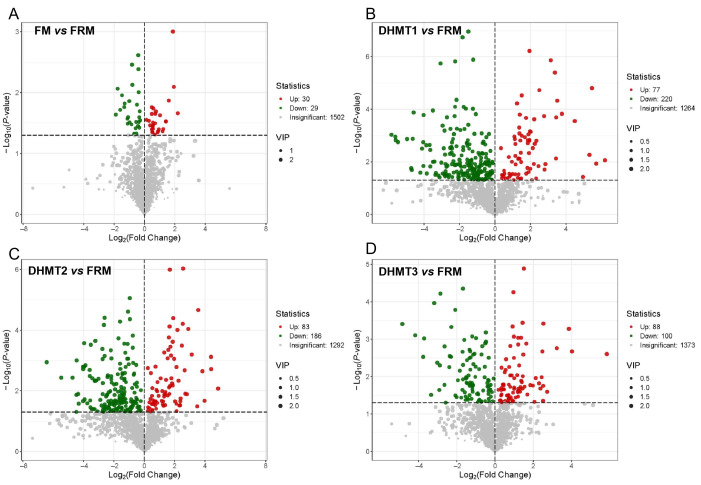
Volcano plot of differential metabolites between different experimental groups (*n* = 5). (**A**) differential metabolites genes between FM and FRM group; (**B**) differential metabolites between DHMT1 and FRM group; (**C**) differential metabolites between DHMT2 and FRM group; (**D**) differential metabolites between DHMT3 and FRM group.Note: FM, fish meal; FRM, fermented rapeseed meal replaced; DHMT1/2/3, FRM + 0.5/1.0/2.0‰ DHM.

The volcano plot analysis of differentially expressed metabolites in the intestine of *P. sinensis* across different comparisons is shown in Figure 8. Compared to the FRM group, a total of 59 differential metabolites were identified in the FM group, comprising 30 upregulated and 29 downregulated metabolites. Following DHM supplementation, 297 differential metabolites were detected in the DHMT1 group relative to the FRM group, including 77 upregulated and 200 downregulated metabolites. In the DHMT2 group, 269 differential metabolites were identified, with 83 upregulated and 186 downregulated. Meanwhile, 188 differential metabolites were observed in the DHMT3 group, consisting of 88 upregulated and 100 downregulated metabolites.

**Figure 9:**
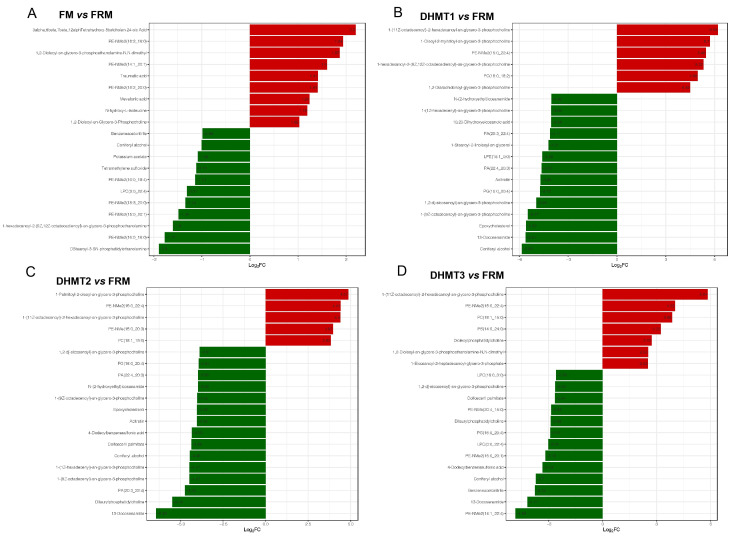
Bar Plot of Fold Change for Top 20 Differential Intestinal Metabolites (*n* = 5). (**A**) Log_2_FC of differential metabolites between FM and FRM group; (**B**) Log_2_FC of differential metabolites between DHMT1 and FRM group; (**C**) Log_2_FC of differential metabolites between DHMT2 and FRM group; (**D**) Log_2_FC of differential metabolites between DHMT3 and FRM group. Note: Red bars represent positive Log_2_FC values, and green bars represent negative Log2FC value. FM, fish meal; FRM, fermented rapeseed meal replaced; DHMT1/2/3, FRM + 0.5/1.0/2.0‰ DHM.

Top 20 differential metabolites in the intestine of *P. sinensis* between FM and FRM groups, DHM supplementation and FRM groups were presented in Figure 9. Overall, the magnitude of change in the FM vs. FRM comparison (|Log_2_FC| ≤ 2.2) was considerably smaller than that observed in the DHM-supplemented groups vs. FRM (|Log_2_FC| ≥ 4.8), indicating that DHM supplementation exerts a more profound regulatory effect on intestinal metabolites. Comparing to FRM group, several metabolites involved in lipid metabolism and bile acid synthesis were significantly upregulated in FM group, including 3alpha,6beta,7beta,12alpha-Tetrahydroxy-5beta-cholan-24-oic Acid, PE-NMe2(18:2_16:0), 1,2-Dioleoyl-sn-glycero-3-phosphoethanolamine-N,N-dimethyl, PE-NMe2(14:1_20:1), Traumatic acid, PE-NMe2(18:2_20:0), Mevalonic acid, N-hydroxy-L-isoleucine, and 1,2-Dioleoyl-sn-Glycero-3-Phosphocholine. In contrast, 11 metabolites were down-regulated, including four phosphatidylethanolamine (PE) derivatives, LPC(0:0_22:4), Benzeneacetonitrile, Coniferyl alcohol, Potassium acetate, Tetramethylene sulfoxide, 1-hexadecanoyl-2-(9Z,12Z-octadecadienoyl)-sn-glycero-3-phosphoethanolamine, and DStearoyl-3-SN-phosphatidylethanolamine.

Compared with the FRM group, the upregulated metabolites in the DHMT1 group included: 1-(11Z-octadecenoyl)-2-hexadecanoyl-sn-glycero-3-phosphocholine, 1-Oleoyl-2-myristoyl-sn-glycero-3-phosphocholine, PE-NMe2(15:0_22:4), 1-hexadecanoyl-2-(9Z,12Z-octadecadienoyl)-sn-glycero-3-phosphocholine, PC(18:0_18:2), and 1,2-Diarachidonoyl-glycero-3-phosphocholine. A total of 14 metabolites were downregulated, including Coniferyl alcohol, 13-Docosenamide, Epoxycholesterol, 1-(9Z-octadecenoyl)-sn-glycero-3-phosphocholine, 1,2-d[Eicosenoyl]-sn-glycero-3-phosphocholine, PG(16:0_20:4), and Acitretin.

Compared with the FRM group, the upregulated metabolites in the DHMT2 group included: 1-Palmitoyl-2-oleoyl-sn-glycero-3-phosphocholine, PE-NMe(15:0_22:4), 1-(11Z-octadecenoyl)-2-hexadecanoyl-sn-glycero-3-phosphocholine, PE-NMe(15:0_20:3), and PC(18:1_15:0). A total of 15 metabolites were downregulated, including 13-Docosenamide, Dilaurylphosphatidylcholine, PA(20:3_22:4), 1-(9Z-octadecenyl)-sn-glycero-3-phosphocholine, 1-(1Z-hexadecenyl)-sn-glycero-3-phosphocholine, and Coniferyl alcohol.

Compared with the FRM group, the upregulated metabolites in the DHMT3 group included: 1-(11Z-octadecenoyl)-2-hexadecanoyl-sn-glycero-3-phosphocholine, PE-NMe2(15:0_22:4), PC(18:1_15:0), PS(14:0_24:0), Dioleoylphosphatidylcholine, 1,2-Dioleoyl-sn-glycero-3-phosphoethanolamine-N,N-dimethyl, and 1-Eicosanoyl-2-heptadecanoyl-glycero-3-phosphate. A total of 14 metabolites were downregulated, including PE-NMe2(14:1_22:4), 13-Docosenamide, Benzeneacetonitrile, Coniferyl alcohol, 4-Dodecylbenzenesulfonic acid, and PE-NMe2(15:0_20:1).

#### 3.6.3. KEGG Enrichment Analysis of Differential Metabolites

**Figure 10:**
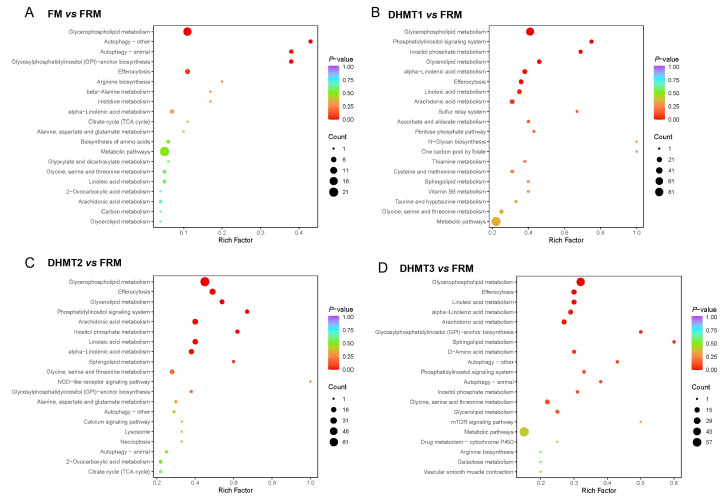
Pathway Enrichment Plot of Differential Intestinal Metabolites (*n* = 5). (**A**) KEGG Enrichment of differential metabolites between FM and FRM group; (**B**) KEGG Enrichment of differential metabolites between DHMT1 and FRM group; (**C**) KEGG Enrichment of differential metabolites between DHMT2 and FRM group; (**D**) KEGG Enrichment of differential metabolites between DHMT3 and FRM group. Note: FM, fish meal; FRM, fermented rapeseed meal replaced; DHMT1/2/3, FRM + 0.5/1.0/2.0‰ DHM.

A KEGG pathway enrichment analysis was performed on the top 20 pathways ranked by *p*-value based on differential metabolite data, as presented in Figure 10. In the FM vs. FRM comparison, the most highly enriched pathway was Glycerophospholipid metabolism, followed by Autophagy-other, Autophagy-animal, Glycosylphosphatidylinositol (GPI)-anchor biosynthesis, and Efferocytosis. Following DHM supplementation, the enriched pathways expanded progressively in a dose-dependent manner. In the DHMT1 vs. FRM comparison, Glycerophospholipid metabolism remained the most enriched pathway, accompanied by the emergence of the Phosphatidylinositol signaling system, Inositol phosphate metabolism, Glycerolipid metabolism, alpha-Linolenic acid metabolism, Efferocytosis, Linoleic acid metabolism, and Arachidonic acid metabolism. In the DHMT2 vs. FRM comparison, differential metabolites were markedly enriched in a broader set of pathways, including Glycerophospholipid metabolism, Efferocytosis, Glycerolipid metabolism, the Phosphatidylinositol signaling system, Arachidonic acid metabolism, Inositol phosphate metabolism, Linoleic acid metabolism, alpha-Linolenic acid metabolism, Sphingolipid metabolism, Glycine, serine and threonine metabolism, the NOD-like receptor signaling pathway, and Glycosylphosphatidylinositol (GPI)-anchor biosynthesis. In the DHMT3 vs. FRM comparison, differential metabolites were prominently enriched in pathways such as Glycerophospholipid metabolism, Efferocytosis, Linoleic acid metabolism, alpha-Linolenic acid metabolism, Arachidonic acid metabolism, Glycosylphosphatidylinositol (GPI)-anchor biosynthesis, Sphingolipid metabolism, D-Amino acid metabolism, Autophagy-other, the Phosphatidylinositol signaling system, Autophagy-animal, Inositol phosphate metabolism, Glycine, serine and threonine metabolism, and Glycerolipid metabolism.

## 4. Discussion

### 4.1. Effects of Dietary DHM on Growth Performance and Digestive Enzyme Activities

Rapeseed meal is not only considerably less expensive than fish meal but also possesses a well-balanced amino acid profile, which is superior to many other commercially available plant protein sources [26,27]. Aside from soybean meal, fermented rapeseed meal, owing to its higher protein content compared to other oilseed meals, represents a promising fish meal alternative and ranks as the second most extensively studied plant protein source to date [3]. In the present study, dietary replacement of 10% fish meal with fermented rapeseed meal (FRM) significantly improved growth performance. Similar growth-promoting effects have also been observed in other aquaculture species, including red sea bream (*Pagrus major*) [28,29] and Pacific white shrimp (*Penaeus vannamei*) [30]. These beneficial effects may be attributable to microbial fermentation, which enhances the production of bioactive substances that improve host digestive capacity [3]. Nevertheless, the observed improvement in growth appears to conflict with the concurrent intestinal mucosal damage induced by FRM. A possible explanation is that the extent of mucosal injury was not severe enough to offset the growth-promoting benefits conferred by the fermented meal.

In this study, dietary supplementation with dihydromyricetin (DHM) significantly improved weight gain rate, specific growth rate, and feed conversion ratio in *P. sinensis*, with the highest dose (2.0‰) yielding the best overall performance. The growth-promoting effects of DHM observed in the present study can be attributed to the improvement of intestinal health and digestive function, which are supported by both our experimental data and existing literature. Based on the digestive enzyme data, DHM supplementation, particularly at medium and high doses, enhanced intestinal digestive enzyme activities, especially trypsin, thereby supporting nutrient digestion and absorption following FRM replacement of FM. Similarly, research on *L. vannamei* demonstrated that dietary DHM (200 mg/kg) significantly enhanced weight gain rate and specific growth rate while reducing feed conversion ratio, accompanied by significantly increased activities of protease, amylase, and lipase [18,24]. In a large yellow croaker (*L. crocea*) trial, final weight, weight gain, survival rate, and specific growth rate were all significantly higher in fish fed DHM at doses ranging from 50 to 300 mg/kg compared to the control group [21]. In contrast, the growth-promoting effects of DHM observed in the present study differ from recent findings in other aquatic species. A study on *M. hoffmanni* reported that dietary supplementation with 0.4‰ DHM did not improve weight gain rate or specific growth rate compared to the control group [22]. Additionally, a study on juvenile hybrid sturgeon (*Acipenser schrenckii* ♀ × *Acipenser baerii* ♂) reported that dietary DHM at 0.3% significantly improved intestinal digestive enzyme activities (protease, amylase, and lipase) and enhanced immune parameters, although the highest dose (0.6%) resulted in decreased growth performance [31]. We propose that the differential effects of DHM on growth performance may be attributed to the following factors: enhancement of digestive enzyme activities; improvement of intestinal structure and enhancement of intestinal antioxidant activity; and species-specific differences.

### 4.2. Intestinal Antioxidant Capacity and Immune Regulatory Function

The present study found that after replacing fishmeal with fermented rapeseed meal (FRM group), the intestinal activities of glutathione (GSH) and catalase (CAT) in *P. sinensis* were significantly reduced compared to the fishmeal control group (FM group), while malondialdehyde (MDA) content decreased significantly to the lowest level in the highest DHM dose group (DHMT3). These findings indicate that FRM substitution induces intestinal oxidative stress, and DHM supplementation can effectively alleviate this effect. The results showing that DHM significantly enhances intestinal GSH and CAT activities in this study are highly consistent with existing literature. Lu et al. also demonstrated in L. vannamei that dietary supplementation with 200 mg/kg DHM significantly increased total antioxidant capacity (T-AOC), antioxidant enzyme activities, and glutathione (GSH) content [18].

In the present study, the levels of pro-inflammatory cytokines IL-1β and TNF-α were significantly elevated in the FRM group, whereas following DHM supplementation, the levels of both cytokines were significantly reduced in all treatment groups. This anti-inflammatory effect is highly consistent with reports demonstrating that DHM inhibits inflammatory signaling pathways in various animal models. In an LPS-induced intestinal injury model in weaned piglets, DHM attenuates lipopolysaccharide-induced intestinal injury in weaned piglets by regulating oxidative stress and inhibiting NLRP3 inflammasome [32], which was consist to our results. In growing-finishing pigs, DHM enhances intestinal antioxidant capacity of growing-finishing pigs by activating ERK/Nrf2/HO-1 signaling pathway [33,34]. The anti-inflammatory cytokine IL-10 exhibited an opposite trend in this study, which is consistent with the findings of Lu et al. in *L. vannamei* [18]. These results indicate that FRM replacement of fishmeal induces intestinal inflammation, and DHM effectively suppresses the expression of pro-inflammatory factors, thereby exerting anti-inflammatory effects.

### 4.3. Intestinal Mucosal Morphology Changes

The integrity of intestinal mucosal morphology is an important indicator for assessing intestinal health and functional status. In this study, H&E staining revealed that the FM group had intact intestinal mucosa with neatly arranged villi and a continuous epithelial layer. In contrast, the FRM group showed obvious mucosal damage, including disorganized villi, reduced villus height, increased lumen space, and mild lamina propria edema. DHM supplementation restored mucosal structure in a dose-dependent manner, with the high-dose DHMT3 group showing significantly higher villus height than the FRM group. These results indicate that DHM effectively repairs intestinal mucosal damage induced by FRM replacement of fishmeal. The results of intestinal mucosal morphology correlated with the levels of intestinal cytokines. Similarly, DHM has demonstrated significant protective potential in terrestrial animal models. It alleviates oxidative stress-induced damage to intestinal epithelial cells [34,35,36]. Furthermore, DHM enhances intestinal barrier function by increasing immune enzyme activities, upregulating the expression and proper distribution of tight junction proteins such as ZO-1, Occludin, and Claudin, as well as modulating the structure of the intestinal microbiota [37].

### 4.4. Intestinal Transcriptome

To date, no study has conducted high-throughput transcriptome sequencing on the intestinal tissues of aquatic animals treated with DHM or vine tea extract. In this study, KEGG enrichment analysis of differentially expressed genes in the intestine of *P. sinensis* revealed dynamic changes in molecular pathways following replacement of fishmeal with fermented rapeseed meal (FRM) and subsequent supplementation with different doses of dihydromyricetin (DHM). The enrichment of structure-related pathways (e.g., cytoskeleton in muscle cells) in the FRM group was consistent with the villus damage and increased lumen space observed in histological analyses, which may reflect a direct impact of FRM the cytoskeletal integrity of intestinal epithelial cells [38]. Meanwhile, the enrichment of pathways related to “intestinal immune network for “IgA production,” “apoptosis,” and “phagosome” suggested that FRM may potentially influence intestinal mucosal immune responses and cellular clearance mechanisms [39], which correlated with the elevated levels of pro-inflammatory cytokines (IL-1β, TNF-α) observed in the FRM group. Low-dose DHM (DHMT1) shifted the enriched pathways toward detoxification metabolism (e.g., drug metabolism-cytochrome P450) and lipid metabolism (e.g., linoleic acid and arachidonic acid metabolism), while immune pathways remained enriched, suggesting a possible association with detoxification and anti-inflammatory programs [40,41,42]. Medium-dose DHM (DHMT2) significantly enriched inflammatory signaling pathways (e.g., cytokine–cytokine receptor interaction, Toll-like receptor signaling, necroptosis) and multiple lipid metabolism pathways (including glycerophospholipid metabolism and various polyunsaturated fatty acid metabolisms), along with the emergence of the ferroptosis pathway. This likely reflects DHM-mediated immune regulation-modulating the cytokine network to shift the immune response from pro-inflammatory toward a reparative phenotype [43]. High-dose DHM (DHMT3) maintained immune regulatory pathways while re-enriching detoxification metabolism pathways and significantly enriching one-carbon metabolism as well as glycine, serine, and threonine metabolism-pathways associated with redox balance. This may be associated with the enhancement of antioxidant defense and metabolic repair capacity by high-dose DHM. One-carbon metabolism provides essential methyl donors and cysteine precursors for glutathione (GSH) synthesis, while glycine, serine, and threonine metabolism directly participates in GSH biosynthesis. These findings may suggest a possible association with the antioxidant phenotype observed in the high-dose DHM group, which exhibited the highest GSH activity and the lowest MDA level. To verify these hypotheses, we examined the transcriptional profiles of representative genes in intestinal tissues. Notably, FRM substitution elicited a marked upregulation of the inflammatory marker *NLRP3* alongside the oxidative detoxification genes *DUOXA2* and *DUOX2*. In contrast, the significantly lower expression levels of these genes in both the DHM-treated and FM groups suggest a comparatively reduced inflammatory status and oxidative stress burden in the intestinal mucosa, indicating that DHM supplementation effectively counteracts the pro-inflammatory and pro-oxidative effects induced by FRM. The significant enrichment of lipid metabolism pathways in this study is consistent with the findings of Lu et al., who reported that DHM significantly downregulated genes involved in fatty acid synthesis (SREBP, FASN, ACC1) and triglyceride synthesis (GPAT, DGAT, PLPP, PLSC), while upregulating genes related to fatty acid oxidation (ACSL, CPT1, ACAD, PAAF), fatty acid transport (FATP), and triglyceride breakdown (HSL, CEL, AGK) in the hepatopancreas of *L. vannamei* using qRT-PCR [18]. These findings are generally consistent with the histological and cytokine results, suggesting a possible transcriptomic link between DHM and the alleviation of FRM-induced intestinal injury, which may involve a synergistic mechanism encompassing detoxification, anti-inflammation, membrane repair, and antioxidant defense.

### 4.5. Intestinal Metabolome

In this study, partial replacement of FM with FRM led to bidirectional changes in intestinal metabolites, mainly involving bile acids, phospholipids, and cholesterol synthesis intermediates. The bile acid is a key regulator of lipid digestion and absorption. Its level in the FRM group was downregulated compared with the FM group, which may reflect an adaptive response of the intestine to lipid absorption from plant protein sources [44]. A decrease in bile acids impairs lipid digestion and absorption and may weaken bile acid-mediated intestinal barrier protection and anti-inflammatory function [45], which is consistent with the observed intestinal mucosal damage and elevated pro-inflammatory cytokine levels in the FRM group. Similarly, fecal and plasma metabolomic analysis revealed bile acids were elevated by DMY administration in dairy cows [46].

Phospholipids (PE-NMe2 series, PC derivatives) are core structural components of cell membranes. The upregulation of various PE derivatives and PC in the FRM group suggests that intestinal epithelial cells may initiate compensatory membrane synthesis in response to FRM-induced membrane damage [47]. Meanwhile, the enrichment of autophagy-related pathways (Autophagy-other, Autophagy-animal) could be linked to the activation of stress survival mechanisms in intestinal cells, possibly in response to anti-nutritional factors or oxidative stress induced by FRM substitution [48]. After DHM intervention, the compensatory upregulation of phospholipids was further enhanced in a dose-dependent manner, particularly in the DHMT2 and DHMT3 groups, which may reflect active membrane repair promotion by DHM, rather than being a mere compensatory response to damage [49]. Moreover, DHM supplementation significantly reduced the enrichment of autophagy pathways, which may be associated with the alleviation of cellular stress burden, potentially contributing to the normalization of autophagic flux and the restoration of intestinal homeostasis.

Compared with the FRM group, the glycerophospholipid metabolism pathway was consistently enriched in FM and all DHM-treated groups, which may suggest a possible association between DHM and the promotion of intestinal epithelial cell membrane synthesis and repair, potentially through the activation of glycerophospholipid metabolism [50]. In other animal models, DHM also prevents hyperlipidemia and diabetes by regulating glycerophospholipid metabolism. For example, in high-fat diet-induced obese mice, DHM effectively prevented hyperlipidemia by affecting glycerophospholipid metabolism, sphingolipid metabolism, and pantothenate/CoA biosynthesis pathways [51]. In a diabetic zebrafish model, integrated metabolomic and transcriptomic analyses revealed that DHM and its hydroxylated derivatives both influence key metabolic pathways, including amino acid metabolism, purine metabolism, and glycerophospholipid metabolism [52].

## 5. Conclusions

Dietary dihydromyricetin (DHM) alleviates fermented rapeseed meal (FRM)-induced intestinal injury in Chinese soft-shelled turtle. FRM substitution caused mucosal damage, elevated pro-inflammatory cytokines (IL-1β, TNF-α), reduced antioxidant enzymes (GSH, CAT), and increased lipid peroxidation (MDA). DHM supplementation (1.0–2.0‰) improved growth performance, restored intestinal morphology, enhanced digestive enzyme activities, and reversed antioxidant and inflammatory imbalances. Integrated multi-omics analysis appears to indicate a potential relationship between DHM treatment and the activation of detoxification pathways (cytochrome P450), upregulation of glycerophospholipid metabolism for membrane repair, and enrichment of polyunsaturated fatty acid metabolism to modulate inflammation. These observations may be associated with the alleviation of FRM-induced intestinal injury, possibly via synergistic mechanisms involving detoxification, anti-inflammation, membrane repair, and antioxidant reinforcement.

## Figures and Tables

**Table 1:** Results of intestinal digestive enzymes and antioxidant indicators of Chinese softshell turtle in different treatments (*n* = 3).

	Values	Treatments				
Items		FM	FRM	DHMT1	DHMT2	DHMT3
Total Protein (g/L)		4.05 ± 0.27	3.46 ± 0.57	3.36 ± 1.09	4.12 ± 0.76	4.88 ± 0.53
Amylase (U/mg prot)		0.07 ± 0.003 ^ab^	0.05 ± 0.007 ^b^	0.09 ± 0.01 ^a^	0.08 ± 0.006 ^ab^	0.06 ± 0.006 ^b^
Trypsin (U/mg prot)		274.26 ± 33.21 ^a^	249.46 ± 27.01 ^a^	267.68 ± 19.75 ^a^	323.19 ± 28.26 ^b^	334.56 ± 27.73 ^b^
GSH (U/mg prot)		195.75 ± 18.00 ^ab^	148.89 ± 11.58 ^b^	236.38 ± 16.76 ^a^	269.49 ± 13.73 ^a^	254.39 ± 12.11 ^a^
CAT (U/mg prot)		123.83 ± 18.05 ^b^	75.03 ± 2.67 ^c^	136.48 ± 11.53 ^b^	265.30 ± 24.44 ^a^	295.57 ± 7.04 ^a^
SOD (U/mg)		60.21 ± 2.52 ^b^	72.98 ± 2.85 ^a^	68.97 ± 4.14 ^ab^	67.51 ± 1.59 ^ab^	74.44 ± 2.27 ^a^
MDA (nmol/mg)		3.90 ± 0.71 ^ab^	10.37 ± 1.97 ^a^	9.09 ± 3.53 ^ab^	7.55 ± 0.80 ^ab^	2.91 ± 0.98 ^b^

Note: FM, fish meal; FRM, fermented rapeseed meal replaced; DHMT1/2/3, FRM + 0.5/1.0/2.0‰ DHM. Different letters in the same row indicate significant differences (*p* < 0.05). The same as below.

## Data Availability

The raw data was uploaded to the National Genomics Data Center (project accession: PRJCA067377; dataset accession: subCRA073822).

## Supplementary Materials

The following supporting information can be downloaded at https://www.mdpi.com/article/10.3390/antiox15070856/s1. Table S1: Experimental Feed Formulation and Main Nutritional Components (Dry Weight). Table S2: Sequences of oligonucleotide primers for qPCR. Table S3: Results of growth performance in different treatments (n = 3). Table S4: Results of transcriptomic sequence assembly of Chinese softshell turtle in different treatments (n = 5). Table S5: Comparison of efficiency statistics of Chinese softshell turtle in different treatments (n = 5). Figure S1: The expression levels of four verified genes were examined by qPCR. Note: different letter means *p* < 0.05. Figure S2: The expression levels of four related genes were examined by qPCR. Note: different letter means *p* < 0.05.
