# Supplementary material for: Dihydromyricetin Alleviates Fermented Rapeseed Meal-Induced Intestinal Injury in Chinese Soft-Shelled Turtle (Pelodiscus sinensis): Insights from Growth, Antioxidant, Inflammatory, Transcriptomic and Metabolomic Assessments
Source: Antioxidants (Basel). 2026 Jul 8;15(7):856. doi: 10.3390/antiox15070856 (PMC13405165; doi:10.3390/antiox15070856)
Supplement: Supplementary file 1 [file antioxidants-15-00856-s001.zip › antioxidants-4367536-supplementary.pdf]

Table S1 Experimental Feed Formulation and Main Nutritional Components (Dry Weight)

Ingredients Percentage (%)	Treatments				
	FM	FRM	DHMT1	DHMT2	DHMT3
Fish meal	50.00	45.00	45.00	45.00	45.00
Fermented rapeseed meal	0.00	8.50	8.50	8.50	8.50
Wheat flour	20.00	20.00	20.00	20.00	20.00
α strach	11.92	8.42	8.37	8.32	8.22
Complex protein	10.50	10.50	10.50	10.50	10.50
Fish oil	2.00	2.00	2.00	2.00	2.00
Soybean oil	1.50	1.50	1.50	1.50	1.50
$Ca(H_2PO_4)_2$	2.00	2.00	2.00	2.00	2.00
Iecithin	0.50	0.50	0.50	0.50	0.50
premixed feed	1.00	1.00	1.00	1.00	1.00
antioxidant	0.01	0.01	0.01	0.01	0.01
Choline	0.50	0.50	0.50	0.50	0.50
VC	0.05	0.05	0.05	0.05	0.05
mould inhibitor	0.02	0.02	0.02	0.02	0.02
DHM	0.00	0.00	0.05	0.10	0.20
Total	100.00	100.00	100.00	100.00	100.00
Moisture	7.50	7.41	7.44	7.39	7.46
Crude protein	41.74	42.82	41.67	42.03	42.15
Crude lipid	6.42	6.65	6.54	6.38	6.27
Crude ash	11.55	11.77	11.61	11.78	11.49

Note: FM, fish meal control; FRM, fermented rapeseed meal replaced; DHMT1/2/3, FRM + 0.5/1.0/2.0‰ DHM. Composite protein contains yeast powder, gluten meal, shrimp meal. The same as below.

Table S2 Sequences of oligonucleotide primers for *q*PCR

Target genes	Primers	Oligonucleotide (5'-3')
β-actin	β-actin-F	ATGGCTCCGGTATGTGCAAA
	β-actin-R	GGCCCATACCAACCATCACA
RBPJL	RBPJL-F	CACTGCGGGAAGGGTACATT
	RBPJL-R	ATGATCTGTGGGGAGGGACA
FBXL22	FBXL22-F	GACTCTGGAAGCTGTGACCC
	FBXL22-R	ATCAGTTTTCCCCCTGGCTG
ITGA5	ITGA5-F	GGCCTGGTGTACGTGTACAA
	ITGA5-R	AAGAAGTCAGGTGAGCTGCC
SYNPO2L	SYNPO2L-F	TCAGGCCGCAAATTCAAAGC
	SYNPO2L-R	GCTCTGTCGCTTGGCAAAAA
NLRP3	NLRP3-F	TGCTCTGTACTGAAGCATCCA
	NLRP3-R	TGCTTCTGTGAACAGGTTAGTGT
CYP8B1	CYP8B1-F	AGAACAGCGCAGAGTTCCTG
	CYP8B1-R	CTTCACAATGGTGCCGAACG
DUOX2	DUOX2-F	ATCGGCAGATATCCCAAGCTCT
	DUOX2-R	CAGCACGACACGTCAAACTT
DUOXA2	DUOXA2-F	GGGCTATAAGAGCGCGGC
	DUOXA2-R	GGCAGGGATCCTGTCCG

Table S3 Results of growth performance in different treatments (n = 3)

Items	FM	FRM	DHMT1	DHMT2	DHMT3
Initial body weight/g	648.75±19.91	639.90±16.93	667.05±4.78	670.28±13.17	648.81±12.43
Final body weigh/g	795.75±20.84^{b}	809.79±25.69^{b}	835.20±5.20^{ab}	865.32±23.46^{a}	876.99±17.08^{a}
Weight gain rate/%	22.69±0.57^{d}	26.51±0.75^{bc}	25.21±0.52^{cd}	29.07±1.29^{b}	35.20±1.88^{a}
Specific growth rate /%/d^{3}	0.37±0.01^{d}	0.42±0.01^{bc}	0.40±0.01^{cd}	0.46±0.02^{b}	0.54±0.02^{a}
Feed conversion ratio	2.22±0.22^{a}	1.95±0.03^{b}	2.35±0.16^{a}	1.88±0.03^{b}	1.69±0.03^{b}
Viscerosomatic index/%	15.11±0.80	15.95±0.87	15.50±0.51	15.59±0.99	16.35±0.73
hepatosomatic index/%	3.75±0.40	3.39±0.34	3.53±0.25	3.18±0.29	4.28±0.48
calipash ratio	3.74±0.14	4.00±0.21	3.93±0.21	3.91±0.16	3.94±0.21

Table S4 Results of transcriptomic sequence assembly of Chinese softshell turtle in different treatments (n = 5)

Sample	Raw Reads	Raw Base (G)	Clean Reads	Clean Base (G)	Error Rate (%)	Q20 (%)	Q30 (%)	GC Content (%)
FM_1	49,537,058	7.43	48,252,660	7.24	0.02	99.53	98.39	47.88
FM_2	52,611,268	7.89	51,325,574	7.70	0.02	99.52	98.38	48.05
FM_3	55,626,844	8.34	54,215,168	8.13	0.02	99.51	98.34	46.87
FM_4	56,540,948	8.48	55,899,744	8.38	0.02	99.52	98.41	45.92
FM_5	50,770,862	7.62	49,722,480	7.46	0.02	99.53	98.46	48.66
FRM_1	50,732,794	7.61	49,824,112	7.47	0.02	99.52	98.34	47.97
FRM_2	49,558,174	7.43	48,133,482	7.22	0.02	99.50	98.31	48.00
FRM_3	63,926,398	9.59	62,662,570	9.40	0.02	99.50	98.27	46.75
FRM_4	55,975,854	8.40	54,738,364	8.21	0.02	99.51	98.35	47.79
FRM_5	53,903,958	8.09	52,586,432	7.89	0.02	99.51	98.33	44.05
DHMT1_1	54,196,620	8.13	52,701,466	7.91	0.02	99.53	98.37	47.26
DHMT1_2	49,554,608	7.43	48,134,520	7.22	0.02	99.51	98.32	48.62
DHMT1_3	55,393,882	8.31	54,249,060	8.14	0.02	99.52	98.36	46.32
DHMT1_4	67,645,086	10.15	65,898,940	9.88	0.02	99.53	98.39	46.69
DHMT1_5	57,664,546	8.65	56,304,698	8.45	0.02	99.54	98.44	46.09
DHMT2_1	57,252,388	8.59	55,363,946	8.30	0.02	99.47	98.21	46.59
DHMT2_2	43,930,048	6.59	43,392,466	6.51	0.02	99.50	98.26	47.63
DHMT2_3	45,858,570	6.88	45,106,286	6.77	0.02	99.53	98.37	47.74
DHMT2_4	66,449,480	9.97	65,310,462	9.80	0.02	99.52	98.34	45.93
DHMT2_5	61,516,798	9.23	60,203,796	9.03	0.02	99.50	98.27	45.82
DHMT3_1	66,795,964	10.02	65,485,424	9.82	0.02	99.52	98.37	46.32
DHMT3_2	47,930,560	7.19	46,529,234	6.98	0.02	99.51	98.35	48.42
DHMT3_3	54,119,014	8.12	52,439,272	7.87	0.02	99.53	98.44	48.39
DHMT3_4	62,461,366	9.37	60,964,120	9.14	0.02	99.48	98.22	46.50
DHMT3_5	51,824,812	7.77	50,487,370	7.57	0.02	99.49	98.24	47.57

Table S5 Comparison of efficiency statistics of Chinese softshell turtle in different treatments (n = 5)

Sample	Total Reads	Reads mapped	Unique mapped	Multi mapped
FM_1	48,252,660	44,457,976 (92.14%)	43,093,355 (89.31%)	1364621(2.83%)
FM_2	51,325,574	47,129,663 (91.82%)	45,637,916 (88.92%)	1491747(2.91%)
FM_3	54,215,168	49,904,395 (92.05%)	48,405,466 (89.28%)	1498929(2.76%)
FM_4	55,899,744	51,861,687 (92.78%)	50,544,805 (90.42%)	1316882(2.36%)
FM_5	49,722,480	45,866,280 (92.24%)	44,389,451 (89.27%)	1476829(2.97%)
FRM_1	49,824,112	46,205,445 (92.74%)	44,693,833 (89.70%)	1511612(3.03%)
FRM_2	48,133,482	44,491,886 (92.43%)	43,106,309 (89.56%)	1385577(2.88%)
FRM_3	62,662,570	58,287,718 (93.02%)	56,721,502 (90.52%)	1566216(2.50%)
FRM_4	54,738,364	49,988,104 (91.32%)	48,454,689 (88.52%)	1533415(2.80%)
FRM_5	52,586,432	49,586,641 (94.30%)	47,622,099 (90.56%)	1964542(3.74%)
DHMT1_1	52,701,466	48,257,699 (91.57%)	46,826,911 (88.85%)	1430788(2.71%)
DHMT1_2	48,134,520	43,208,920 (89.77%)	41,891,537 (87.03%)	1317383(2.74%)
DHMT1_3	54,249,060	50,096,995 (92.35%)	48,613,477 (89.61%)	1483518(2.73%)
DHMT1_4	65,898,940	60,816,633 (92.29%)	59,171,715 (89.79%)	1644918(2.50%)
DHMT1_5	56,304,698	51,959,399 (92.28%)	50,389,560 (89.49%)	1569839(2.79%)
DHMT2_1	55,363,946	51,530,657 (93.08%)	50,085,416 (90.47%)	1445241(2.61%)
DHMT2_2	43,392,466	40,308,448 (92.89%)	39,034,298 (89.96%)	1274150(2.94%)
DHMT2_3	45,106,286	40,826,126 (90.51%)	39,497,031 (87.56%)	1329095(2.95%)
DHMT2_4	65,310,462	59,440,474 (91.01%)	57,777,177 (88.47%)	1663297(2.55%)
DHMT2_5	60,203,796	55,254,914 (91.78%)	53,783,578 (89.34%)	1471336(2.44%)
DHMT3_1	65,485,424	56,777,539 (86.70%)	55,287,369 (84.43%)	1490170(2.28%)
DHMT3_2	46,529,234	42,567,778 (91.49%)	41,208,596 (88.56%)	1359182(2.92%)
DHMT3_3	52,439,272	46,499,676 (88.67%)	44,987,506 (85.79%)	1512170(2.88%)
DHMT3_4	60,964,120	56,659,049 (92.94%)	55,042,258 (90.29%)	1616791(2.65%)
DHMT3_5	50,487,370	46,588,220 (92.28%)	45,200,253 (89.53%)	1387967(2.75%)

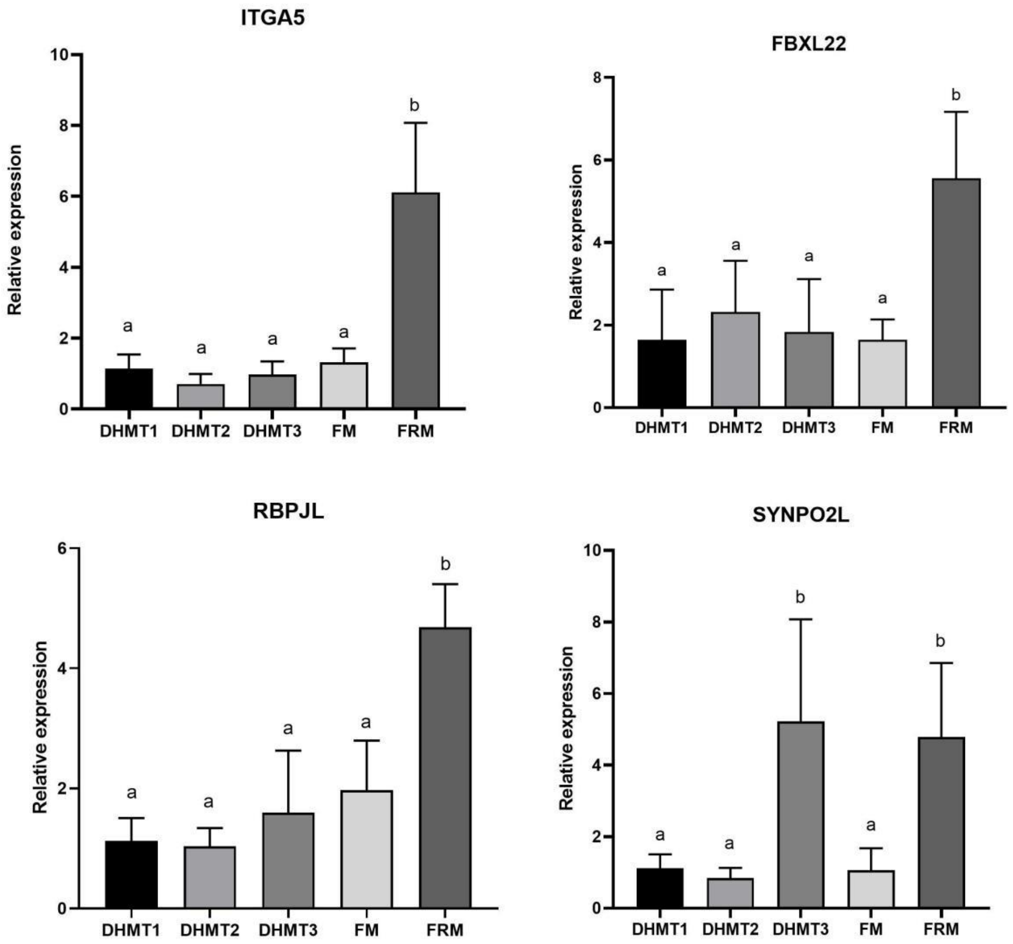

Figure S1. The expression levels of four verified genes were examined by qPCR. Note: different letter means $p < 0.05$.

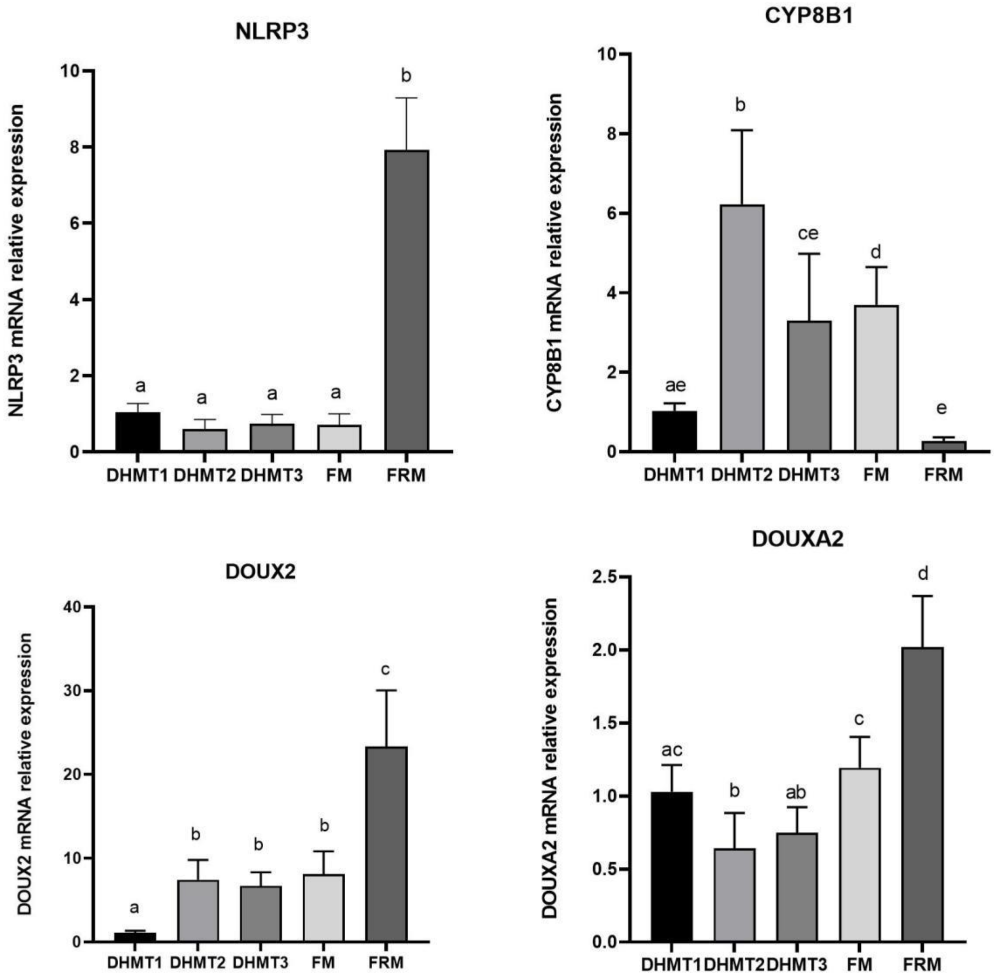

Figure S2. The expression levels of four related genes were examined by *q*PCR. Note: different letter means $p < 0.05$.
